# Self-correction in biomedical publications and the scientific impact

**DOI:** 10.3325/cmj.2014.55.61

**Published:** 2014-02

**Authors:** Armen Yuri Gasparyan, Lilit Ayvazyan, Nurbek A. Akazhanov, George D. Kitas

**Affiliations:** 1 Departments of Rheumatology and Research & Development, Dudley Group NHS Foundation Trust (Teaching Trust of University of Birmingham), Russells Hall Hospital, Dudley, United Kingdom; 2 Department of Medical Chemistry, Yerevan State Medical University, Yerevan, Armenia; 3 Department of Internship and Residency for General Practitioners N3, Kazakh National Medical University, Almaty, Kazakhstan; 4 Arthritis Research UK Epidemiology Unit, University of Manchester, Manchester, United Kingdom

## Abstract

**Aim:**

To analyze mistakes and misconduct in multidisciplinary and specialized biomedical journals.

**Methods:**

We conducted searches through PubMed to retrieve errata, duplicate, and retracted publications (as of January 30, 2014). To analyze publication activity and citation profiles of countries, multidisciplinary, and specialized biomedical journals, we referred to the latest data from the SCImago Journal & Country Rank database. Total number of indexed articles and values of the *h*-index of the fifty most productive countries and multidisciplinary journals were recorded and linked to the number of duplicate and retracted publications in PubMed.

**Results:**

Our analysis found 2597 correction items. A striking increase in the number of corrections appeared in 2013, which is mainly due to 871 (85.3%) corrections from *PLOS One*. The number of duplicate publications was 1086. Articles frequently published in duplicate were reviews (15.6%), original studies (12.6%), and case reports (7.6%), whereas top three retracted articles were original studies (10.1%), randomized trials (8.8%), and reviews (7%). A strong association existed between the total number of publications across countries and duplicate (r_s_ = 0.86, *P* < 0.001) and retracted items (r_s_ = 0.812, *P* < 0.001). A similar trend was found between country-based *h*-index values and duplicate and retracted publications.

**Conclusion:**

The study suggests that the intensified self-correction in biomedicine is due to the attention of readers and authors, who spot errors in their hub of evidence-based information. Digitization and open access confound the staggering increase in correction notices and retractions.

In biomedicine, PubMed is the largest, most informative, and well-organized search platform, which retrieves sources from the MEDLINE database and PubMed Central (PMC) digital archive. It contains more than 23 million records, including 2.7 million references from PMC. All citations to MEDLINE-indexed sources are tracked by Scopus and influence the impact indicators in the SCImago Journal & Country Rank portal. Optimal searches through PubMed imply the use of the terms listed in the Medical Subject Headings (MeSH) controlled vocabulary (1).

The MeSH vocabulary registered the following terms/publication types, which can be entered into PubMed searches for analyzing mistakes and misconduct in biomedical publications: published erratum (or errata, or corrigenda; first introduced in 1991), duplicate publication (1991), and retracted publication (1989).

An erratum is published by an author, editor, or publisher, when a minor, correctable mistake is noticed in the author by-lines, text, or graphical material of an index publication. The validity of conclusions in the index publication is not questioned, and its content remains unchanged, with a link to the published errata in PubMed. Authors themselves are usually responsible for minor mistakes. The reviewers’ oversight, “soft” attitude throughout the review process, automatic formatting, and superficial proofreading may all lead to incorrect publications. The reasons of mistakes are mentioned in the errata.

Duplicate, or multiple, or redundant publication is a form of academic misconduct, resulting from dual or multiple submissions of a nearly identical or identical work to several journals. Duplicates do not inform reviewers, editors, and publishers about previous submission or publication of the same work. Delays with processing manuscripts in some journals and lack of proper guidance in the instructions to authors may be the reasons of duplication committed by some inexperienced authors. This is why some editors formally urge their authors to submit exclusive, original material with “high scientific and ethical quality” (2). Overlapping manuscripts can be products of self-plagiarism, salami slicing, and augmentation of previous publications (2-4). Overlapping scientific materials overburden the reviewers’ time and confuse processing of scientific evidence in systematic reviews and meta-analyses (5,6). Duplicate articles sometimes violate the publishers’ copyrights and require retractions. The complexity of forms of covert duplication on the one hand, and deficient cross-indexing of duplicates in bibliographic databases on the other make it impossible to reveal the real frequency of the misconduct. Notices of duplication are often published in PubMed, but are not properly anchored by proper MeSH terms and do not always disclose the origins of the duplication. Duplicates beyond the PubMed platform, including non-English ones, are not tracked. Even simultaneous, ethical publication of practice guidelines in several journals is not always properly documented, making it difficult to distinguish unethical duplication from acceptable secondary publication.

Retracted publications are better represented in PubMed and designated as separate publication types in the MeSH hierarchy. Unless there is a copyright violation, each of the retracted items remains on the publisher’s website with a clear mark of the withdrawal from press. A retraction of publication is a freely available, indexed note, which can be issued by authors, editors, or publishers of the index publication to state the reasons of retraction and to avoid further reliance on and citations of poor quality and potentially harmful for health care sources (7). Retractions are issued because of fraud, inconsistent reporting, mistakes, plagiarism, duplication, legal (copyrights violation) and ethical concerns (absence of institutional approval), disputed authorship, and production errors (8).

Analyzing updates on corrections, duplicate, and retracted publications in PubMed is an exercise for authors, reviewers, and editors, which may help avoid future mistakes at various stages of scientific research and publishing. We therefore aimed to analyze mistakes and misconduct in multidisciplinary and specialized biomedical journals.

## Methods

We conducted searches through PubMed to retrieve errata, duplicate, and retracted publications (as of January 30, 2014). Using PubMed filters, we recorded most frequent duplicate and retracted article types. To analyze publication activity and citation profiles of countries, multidisciplinary, and specialized biomedical journals, we referred to the latest data from the SCImago Journal & Country Rank database. Total number of indexed articles and values of the *h*-index of the fifty most productive countries and multidisciplinary journals were recorded and linked to the number of duplicate and retracted publications in PubMed.

For analysis of the scope and the extent of duplicate and retracted publications in specialized journals, we chose rheumatology, a rapidly developing field of science, where the number of articles has tripled over the past two decades (9). Thirty-seven rheumatology journals, visible in PubMed and ranked in the SCImago database, were chosen to analyze publications profile. The Journal Impact Factor (JIF) values of rheumatology journals, indexed in PubMed, were recorded from Thomson Reuters’ Journal Citation Reports^®^ (2013 edition). All cases of retracted rheumatological articles were analyzed in detail.

### Statistical analysis

The data are presented as frequencies and relative frequencies. Number of articles and impact indicators were tested for normality by the Shapiro-Wilk test, and Spearman’s rank correlation analyses were employed to assess the relationships between the variables. *P* values below 0.05 were considered statistically significant. The data were analyzed using IBM SPSS, version 20 software (IBM SPSS Inc, Chicago, IL, USA).

## Results

In PubMed, there were 2597 correction items (as of January 30, 2014), with the first one published in 1978. There were two annual peaks – the first one with 148 corrections (6%) in 1995 and the second one with 1021 (39%) in 2013. The striking increase in corrections in 2013 was largely due to 871 (85%) corrections from *PLOS One*. The journal published 1224 (47%) corrections so far.

There were 1086 duplicate publications indexed in PubMed. The first duplicate article was indexed in 1969, and relatively large numbers of duplicates were recorded in 2003 (n = 74) and in 2012 (n = 77). Employing several PubMed search filters, we found that articles mostly published in duplicate were reviews, comparative original studies, and case reports (Table 1). Of the 1086 duplicate items, only 14 (1%) were currently tagged as retracted due to various reasons.

**Figure 1:**
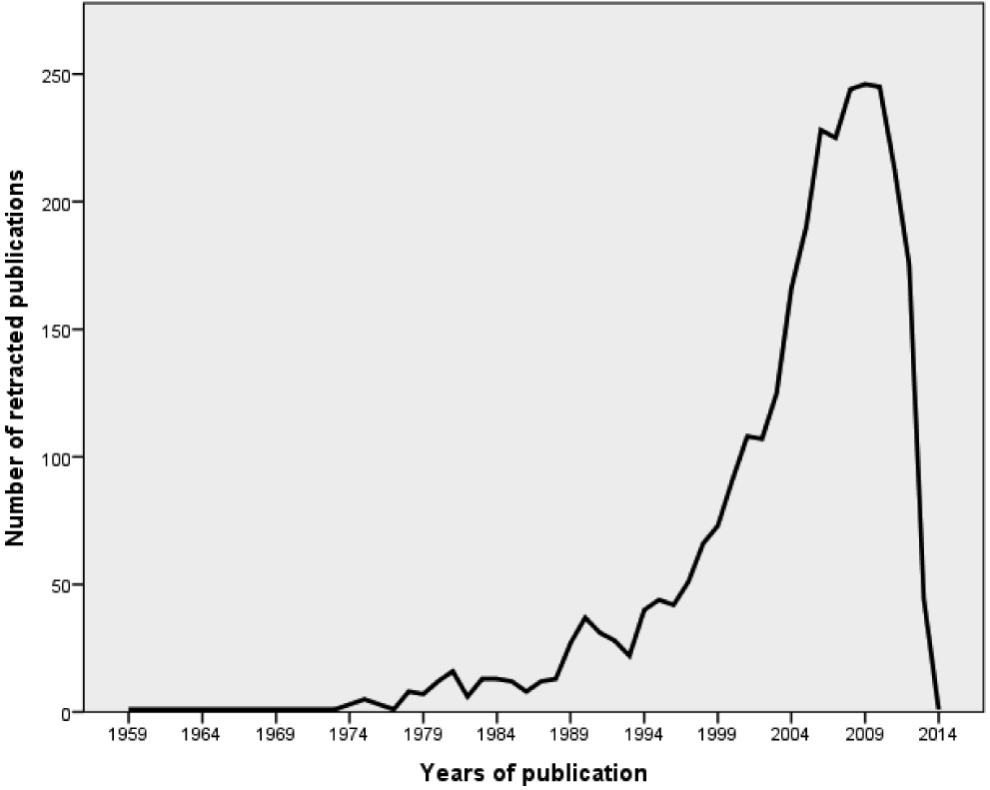
The number of retracted publications in PubMed (as of January 30, 2014)

The record of retracted items in PubMed has already reached 3000. The first paper was published in 1959, and the largest number (246) was recorded in 2009 (Figure 1). Notably, 2414 (80%) of the retracted items were published from January 1, 2000 to December 31, 2013, the period of proactive open-access movement and digitization of periodicals. The top three retracted article types were comparative studies, randomized trials, and reviews (Table 1).

**Figure 2:**
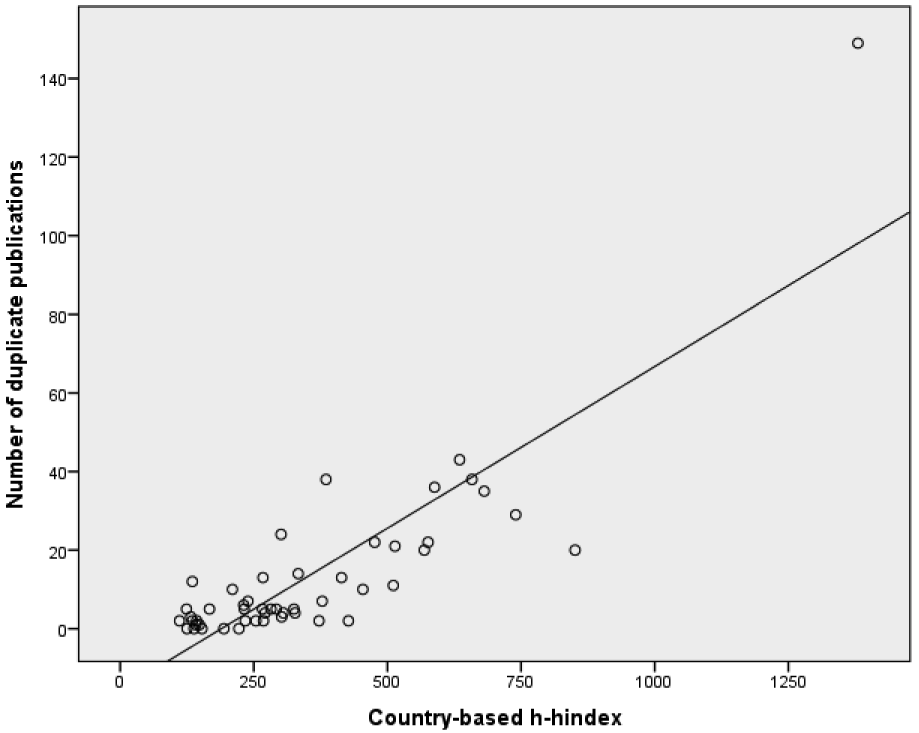
Correlation between the number of duplicate items and the *h*-index values (r_s_ = 0.74, *P* < 0.001)

**Figure 3:**
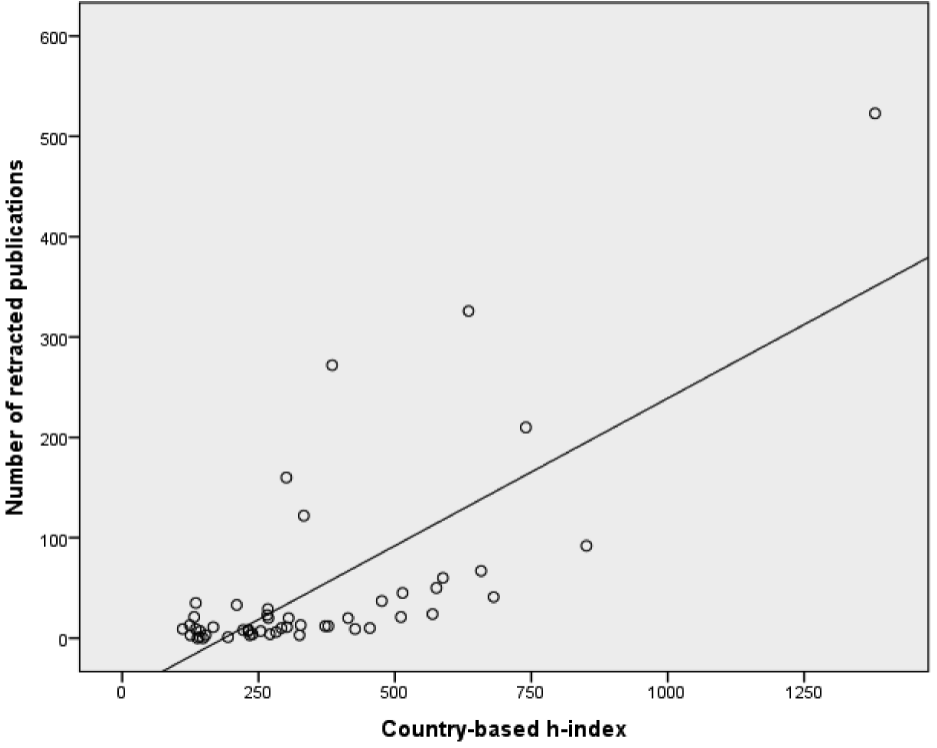
Correlation between the number of retracted items and country-based *h*-index values (r_s_ = 0.66, *P* < 0.001)

Comprehensive analysis of country profiles in the SCImago Journal & Country Rank database and PubMed revealed that the United States leads in terms of duplicate and retracted items (Table 2). The list of countries with more than 100 retractions includes the United States (523 [17%]), Japan (326 [11%]), China (272 [9%]), Germany (210 [7%]), India (160 [5%]), and South Korea (122 [4%]). Correlations between country-based impact indicators and publication records showed a significant association of total number of publications with duplicate (r_s_ = 0.86, *P* < 0.001) and retracted items (r_s_ = 0.81, *P* < 0.001). A similar trend was found between country-based *h*-index, the integrative metric of productivity and citations, and duplicate (r_s_ = 0.74, *P* < 0.001) and retracted publications (r_s_ = 0.66, *P* < 0.001) (Figures 2 and 3).

**Figure 4:**
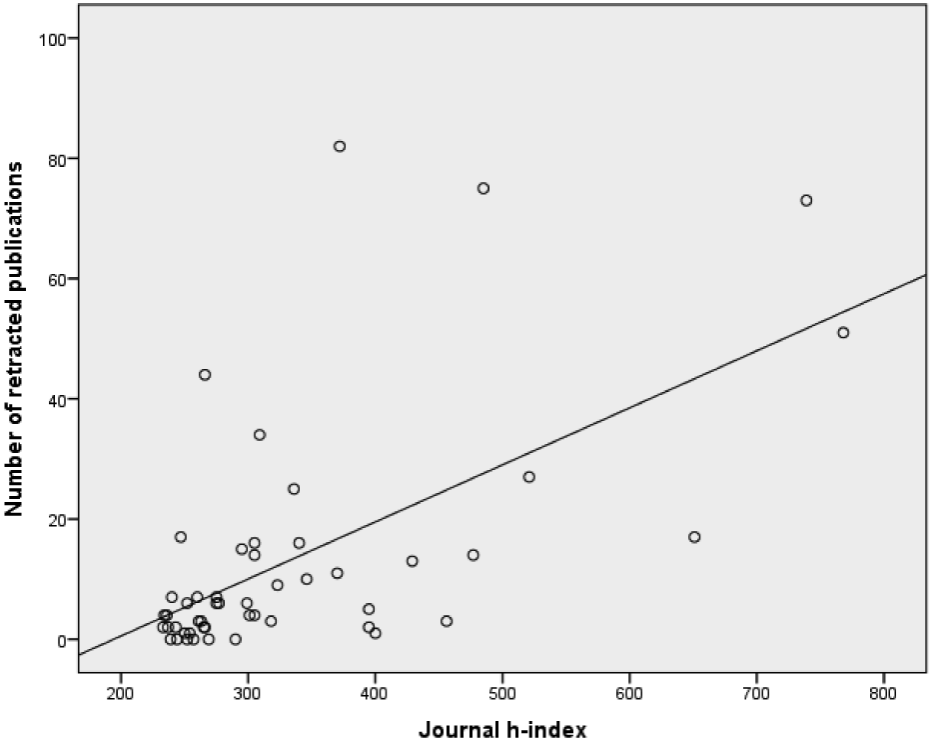
Correlation between the number of retracted publications and top journals’ *h*-index values (r_s_ = 0.56, *P* < 0.001)

The fifty top-tier journals’ *h*-index values were analyzed in the context of their quantitative records of duplicate and retracted items (Table 3). The journal *h*-index and number of duplicate publications were not associated (r_s_ = 0.07, *P* = 0.647), suggesting that these items are scattered across a larger number of PubMed-indexed journals, irrespective of the impact indicators. Retracted publications were largely found in the top-tier journals such as the *Journal of Biological Chemistry* (82 articles), *Proceedings of the National Academy of Sciences of the USA* (75 items), *Science* (73 items), *Nature* (51 items), *Journal of Immunology* (44 items), *Blood* (34 items), *Cell* (27 items), *Journal of Clinical Investigation* (25 items), *New England Journal of Medicine* (17 items), and *Molecular and Cellular Biology* (17 items). There was a significant, though not strictly linear correlation between the number of retracted items and journal *h*-index values of the top journals ranked in Table 3 (r_s_ = 0.56, *P* < 0.001; Figure 4). The only journal with a strikingly large number of retractions, which is not listed in Table 3, is *Anesthesia & Analgesia* (63 items), with the *h*-index of 130 being ranked in the 329 place in the SCImago database.

Quantitative records of duplicate and retracted items of the thirty-seven rheumatology journals visible in PubMed and listed in the SCImago database are presented in Table 4. Only six items of these journals were tagged as duplicates (10-15). Of these, one editorial from the USA was a simultaneous, ethically acceptable publication in *Arthritis & Rheumatism* (10). The second duplicate item, which was published in the *Annals of the Rheumatic Diseases* in 1995, overlapped substantially with a similar PubMed-indexed original research paper by Italian authors (11). An informative notice of redundant publication and violation of the authorship norms appeared in the same journal the following year. Two identical meta-analyses from South Korea were published in the same issue of *Rheumatology International* in 2006, possibly due to production mistakes (12,13). Finally, two overlapping practice guidelines in Croatian were published in *Reumatizam* in 2007 and 2008 (14), and commented on in an erratum in 2009. The twenty-two retracted articles were unevenly distributed across rheumatology journals (16-37).

No association between the number of retractions and the impact indicators of rheumatology journals was revealed. *Arthritis & Rheumatism*, *Clinical Rheumatology, and Rheumatology International* altogether retracted 14 items (63.6%). Details of the indexed items and their notices of retraction pinpoint to a diversity of article types, geography, and reasons of retractions (Table 5). Articles published in 2001-2012 were retracted in 2006-2013, suggesting that the retraction is relatively new to rheumatology. Retracted research papers in *Arthritis & Rheumatism* attracted the largest number of citations in Scopus. Unintentional mistakes and inappropriate statistical analyses were common reasons for retractions in top rheumatology journals such as *Arthritis & Rheumatism* and *Rheumatology (Oxford)*. Seven out of the total 22 retractions (32%) were due to plagiarism in reviews, which were published in lower-impact periodicals such as *Clinical Rheumatology* and *Journal of Clinical Rheumatology*. Thirteen retracted articles (59%) came from the same authors (16,17,25-28,30-36), who repetitively published erroneous and/or unethical research reports. As a prime example, Bernardino Saccomanni, listed in PubMed and Scopus as Saccomanni B and Berdardino S, from Orthopedic and Traumatologic Surgery, Bari, Italy, published four reviews in *Clinical Rheumatology* (Springer) and one in *Rheumatology International* (Springer), which were all retracted due to substantive plagiarism. Importantly, the same publisher retracted five items of this serial plagiarist in *Current Reviews in Musculoskeletal Medicine* (orthopedics and sports medicine subject category), one in *Musculoskeletal Surgery* (Medicine), one in *Osteoporosis International* (Medicine), and one in *Knee Surgery, Sports Traumatology, Arthroscopy* (orthopedics and sports medicine). In all retracted reviews, Bernardino Saccomanni was the sole author.

## Discussion

A recent report on the frequency of errata claimed that “the proportion of errata has remained relatively constant since the 1970s” (38). However, our analysis proved that the publication of these notes boosted in 2013, mostly because of self-corrections in *PLOS One*. This relatively new and large open-access journal appears to have adopted a “publish first, judge later” publishing model (39). Our searches also revealed an increase of duplicate items, which mostly came to the light in the digitization and open-access era. This bit of information prompts “busy” reviewers and editors to be more vigilant and check certain types of submissions for overlaps and text recycling.

Research environment and publishing activity in different countries seem to be critical factors of erroneous, duplicate, and unethical publishing. Our analysis, based on the author information of duplicate and retracted publications in PubMed, reveals that a sizeable amount of these items come from highly productive countries. No country or journal is immune to research misconduct and publication of incorrect, falsified, and misleading information. And the initial step toward avoiding the misconduct is to widely distribute published sources and reach out to skilled readers, who can spot minor and major mistakes. Not surprisingly, widely-read top-tier journals (eg, *Journal of Biological Chemistry, Science, and Nature*) and periodicals employing the open-access publishing model (eg, *PLOS One*) often retract or publish corrections. Moreover, time from publication to retraction of sources in the highest-impact journals is shorter than in those with lower impact, presumably due to the lack of attention of readers and authors to the latter ones (40). Shorter time-to-retraction is particularly evident from 2002 onwards (40), the period of proactive open-access movement (41).

The notorious serial case of plagiarism by Bernardino Saccomanni suggests that the true extent of retractions in certain areas cannot be judged by focusing on a single subject category. Erroneous rheumatological publications are found in a range of journals covering issues in general and internal medicine, immunology, pain management, rehabilitation and physical therapy, and pharmacotherapy. Employing the commonly used term – “antirheumatic agents,” we retrieved 19 additional retracted publications from various “non-rheumatology” journals, which were not listed in Table 5.

Inconsistencies, redundancies, unintentional mistakes, errors, and apparently misleading information are deeply rooted in biomedical literature. Uncovering the complexity and true extent of the research and publishing activities damaging the science communication is still in its early stage. The global digitization, open access, and switching to English as a universal language of scientific communication help the whole system to self-clean and enhance reporting. The PubMed search platform, interconnected with MEDLINE and MeSH thesaurus, remains the only well-organized and informative tool for analyzing erroneous and unethical biomedical reports, which should be left out at the evidence processing for systematic reviews and meta-analyses (42). Scopus and SCImago Journal & Country Rank citation-tracking and impact-calculating databases complement PubMed by quantifying citations and overall impact of errors and misconduct.

We are still unaware how many flawed items have been published in journals not covered by PubMed. The number of journals being accepted for indexing in PubMed and archiving in PubMed Central is constantly growing, which may increase the number of corrections, detected duplications, and retractions (43).

Editors and publishers should regularly analyze in-house mistakes and oversights, which lead to erroneous publications, and open access to the index sources, accompanying them with explicit and free-to-read notices of corrections, duplications, or retractions. Such notices, indexed by various bibliographic databases, can serve educational tools for future authors and become research material, which is currently robust enough for retractions research only (44,45).

Peer review is one of the pillars of scholarly publishing, which is meant to prevent the evidence accumulation from the influx of unreliable and poor quality research data. Peer reviewers and editors bear their share of responsibility for selecting scientifically sound research reports. Their research experience from mainstream science countries and statistical skills often make them suitable for the reviewing and editorial work (46,47). To date, no study examined the role of reviewers in withdrawing redundant, plagiarized, or falsified journal submissions. It is also unknown whether the reviewers ever requested corrections or retractions post-publication. Interestingly, a study of 312 retractions from 187 journals, indexed in MEDLINE from 1988 to 2008, revealed that editors themselves issued 65 (21%) retraction notices (48). Based on our analyses, reviewers and responsible editors may play a more active role in the rejection of retractable submissions when the authors’ identity is known to them (single-blind or public peer review), online links to the authors’ profile in PubMed are activated, a statistical reviewer is involved in the analysis of original research, and software is employed to check for duplicate submissions and text recycling in reviews (eg, CrossCheck^TM^ powered by iThenticate^®^) (49). Our analysis of multidisciplinary and rheumatology journals, coupled with the results of a few recent studies (50,51), prompts future reviewers to be more cautious when evaluating randomized control trials and reviews, particularly from certain countries (eg, USA, Japan, China) and subject areas with high impact (drug therapy).

Reviewers, editors, and other stakeholders of science communication should be aware how the system undergoes self-cleaning. The guidance from editorial associations and expert discussions at some online forums are the current tools for avoiding or retracting unwanted publications. The International Committee of Medical Journal Editors (ICMJE) clearly distinguished ethically acceptable secondary publications from redundancies and set rules for judging competing and complementary manuscripts (52). A guidance on how to issue corrections, “expressions of concern,” and retractions is also provided by the ICMJE (53). In case of suspected misconduct, editors are advised to follow the guidance from the Committee on Publication Ethics (COPE). COPE published flowcharts for editors on what to do when redundant, plagiarized, or fabricated publications are suspected, which are also of interest to authors and reviewers (54-56). Additionally, COPE experts presented an action plan for editors confronted with the absence of patients’ informed consent, violation of institutional ethics restrictions, nondisclosure of conflicts of interest, and disputed authorship, all of which may require appropriate corrections or retractions (7). Member-restricted discussions of a series of fraudulent articles are discussed at COPE to uncover reasons of misconduct and provide a message for all stakeholders of science communication. Discussions are also available at the Retraction Watch blog (*http://retractionwatch.com/*), which was launched in 2010. Since then, blogging on retractions has been recognized as a valuable source of information by scientists worldwide. Motto of the Retraction Watch is “tracking retractions as a window into the scientific process.” Since the launch, new cases of retractions have been selectively picked and announced to the public, thus encouraging open discussion of the authors’ unacceptable behavior and the publishers’ actions to prevent future retractions. A large number of cases of plagiarism, fraud, and retractions, which are commented on at the blog, relate to biomedicine.

## 

**Table 1:** Duplicate and retracted articles in PubMed (as of January 30, 2014)

Article types	Duplicate items, No. (%)	Retracted items, No. (%)
Case reports	83 (7.6)	90 (3.0)
Comparative studies	137 (12.6)	302 (10.1)
Randomized controlled trials	57 (5.2)	263 (8.8)
Systematic reviews	65 (6.0)	38 (1.3)
Meta-analyses	4 (0.4)	14 (0.5)
Reviews	170 (15.6)	210 (7.0)
Editorials	45 (4.1)	10 (0.3)
Practice guidelines	10 (0.9)	8 (0.3)
Letters	38 (3.5)	48 (1.6)
News items	0 (0)	3 (0.1)
Non-English sources	74 (6.8)	72 (2.4)
US NIH supported sources	4 (0.4)	5 (0.2)
Total	1086 (100)	3000 (100)

**Table 2:** Impact indicators, duplicate, and retracted publications of the fifty most productive countries*

Rank	Country	Total No. of articles	*h* index	Duplicate items, No. (%)	Retracted items, No. (%)
1	United States	7,063,329	1,380	149 (13.7)	523 (17.4)
2	China	2,680,395	385	38(3.5)	272 (9.1)
3	United Kingdom	1,918,650	851	20 (1.8)	92 (3.1)
4	Germany	1,782,920	740	29 (2.7)	210 (7.0)
5	Japan	1,776,473	635	43 (4.0)	326 (10.9)
6	France	1,283,370	681	35 (3.2)	41 (1.4)
7	Canada	993,461	658	38 (3.5)	67 (2.2)
8	Italy	959,688	588	36 (3.3)	60 (2.0)
9	Spain	759,811	476	22 (2.0)	37 (1.2)
10	India	750,777	301	24 (2.2)	160 (5.3)
11	Australia	683,585	514	21 (1.9)	45 (1.5)
12	Russian Federation	586,646	325	5 (0.4)	3 (0.1)
13	South Korea	578,625	333	14 (1.3)	122 (4.1)
14	Netherlands	547,634	576	22 (2.0)	50 (1.7)
15	Brazil	461,118	305	4 (0.4)	20 (0.7)
16	Taiwan	398,720	267	13 (1.2)	29 (1.0)
17	Switzerland	395,703	569	20 (1.8)	24 (0.8)
18	Sweden	375,891	511	11 (1.0)	21 (0.7)
19	Poland	346,611	302	3 (0.3)	11 (0.4)
20	Turkey	306,926	210	10 (0.9)	33 (1.1)
21	Belgium	299,077	454	10 (0.9)	10 (0.3)
22	Israel	224,674	414	13 (1.2)	20 (0.7)
23	Austria	214,844	378	7 (0.6)	12 (0.4)
24	Denmark	208,227	427	2 (0.2)	9 (0.3)
25	Iran	202,807	135	12 (1.1)	35 (1.2)
26	Finland	190,192	372	2 (0.2)	12 (0.4)
27	Greece	180,688	266	5 (0.4)	23 (0.8)
28	Mexico	166,604	232	5 (0.4)	8 (0.3)
29	Czech Republic	163,740	239	7 (0.6)	4 (0.1)
30	Hong Kong	162,812	292	5 (0.4)	10 (0.3)
31	Norway	162,390	327	4 (0.4)	13 (0.4)
32	Singapore	149,509	268	2 (0.2)	20 (0.7)
33	Portugal	138,892	234	2 (0.2)	3 (0.1)
34	New Zealand	129,822	282	5 (0.4)	6 (0.2)
35	South Africa	125,303	231	6 (0.5)	7 (0.2)
36	Argentina	118,347	222	0 (0)	8 (0.3)
37	Hungary	112,177	254	2 (0.2)	7 (0.2)
38	Ukraine	110,291	142	1 (0.09)	1 (0.03)
39	Ireland	104,634	271	4 (0.4)	4 (0.1)
40	Malaysia	99,187	125	0 (0)	3 (0.1)
41	Romania	92,264	135	2 (0.2)	9 (0.3)
42	Egypt	89,489	132	3 (0.3)	21 (0.7)
43	Thailand	82,209	167	5 (0.4)	11 (0.4)
44	Chile	68,974	194	0 (0)	1 (0.03)
45	Saudi Arabia	58,840	124	5 (0.4)	13 (0.4)
46	Pakistan	58,133	111	2 (0.2)	9 (0.3)
47	Croatia	57,454	143	2 (0.2)	7 (0.2)
48	Slovakia	56,552	148	1 (0.09)	0 (0)
49	Slovenia	50,565	153	0 (0)	3 (0.1)
50	Bulgaria	45,348	138	0 (0)	0 (0)

*The number of documents and the *h*-index values are obtained from the SCImago Journal & Country Rank Database, where 238 countries are listed. The number of duplicate and retracted items is retrieved from PubMed, based on corresponding author information. The percentages are calculated in relation to 1086 total duplicates and 3000 retractions (as of January 30, 2014).

**Table 3:** Journal *h*-index values and number of duplicate and retracted items in top-tier journals*

Rank	Journal abbreviations	Journal h-index	Duplicate items, No.	Retracted items, No.
1	*Nature*	768	0	51
2	*Science*	739	0	73
3	*N Engl J Med*	651	1	17
4	*Cell*	521	0	27
5	*Proc Natl Acad Sci U S A*	485	1	75
6	*Lancet*	477	4	14
7	*JAMA*	456	3	3
8	*Circulation*	429	8	13
9	*Chem Rev*	400	0	1
10	*Nat Genet*	395	0	2
11	*Phys Rev Lett*	395	0	5
12	*J Biol Chem*	372	1	82
13	*Nat Med*	370	0	11
14	*J Clin Oncol*	346	2	10
15	*J Am Chem Soc*	340	0	16
16	*J Clin Invest*	336	0	25
17	*J Exp Med*	323	0	9
18	*Genes Dev*	318	0	3
19	*Blood*	309	0	34
20	*Angew Chem Int Ed Engl*	305	0	4
21	*Cancer Res*	305	0	16
22	*J Neurosci*	305	0	14
23	*Neuron*	301	0	4
24	*Nucleic Acids Res*	299	2	6
25	*EMBO J*	295	0	15
26	*Appl Phys Lett*	290	0	0
27	*J Am Coll Cardiol*	277	2	6
28	*BMJ*	275	4	6
29	*J Cell Biol*	275	0	7
30	*Phys Rev B Condens Matter Mater Phys*	269	0	0
31	*Gastroenterology*	266	2	2
32	*J Immunol*	266	1	44
33	*Nat Biotechnol*	265	0	2
34	*Ann Intern Med*	263	0	3
35	*Mol Cell*	261	0	3
36	*Immunity*	260	0	7
37	*Nat Neurosci*	257	0	0
38	*J Natl Cancer Inst*	254	0	1
39	*Am J Respir Crit Care Med*	252	2	6
40	*Nat Rev Mol Cell Biol*	252	0	0
41	*J Phys Chem B*	250	0	1
42	*Mol Cell Biol*	247	0	17
43	*Nat Rev Cancer*	244	0	0
44	*Nat Immunol*	243	0	2
45	*Neurology*	240	16	7
46	*Nano Lett*	239	0	0
47	*Am J Psychiatry*	237	0	2
48	*Nat Cell Biol*	236	0	4
49	*J Clin Endocrinol Metab*	234	3	4
50	*Hepatology*	233	1	2

*The selected journals are ranked according to their *h*-index in the SCImago Journal & Country Rank Database, where 20 544 multidisciplinary journals are listed (as of January 30, 2014). The records of duplicate and retracted items of the most-impacting journals are retrieved from PubMed.

**Table 4:** Duplicate and retracted publications in rheumatology journals

Rank	Journal abbreviations	Journal h-index	2-Y JIF	Duplicate items, No.	Retracted items, No.
1	*Arthritis Rheum*	211	7.477	**1**	**5**
2	*Ann Rheum Dis*	132	9.111	**1**	**2**
3	*J Rheumatol*	124	3.258	0	0
4	*Rheumatology (Oxford)*	106	4.212	0	**1**
5	*Osteoarthritis Cartilage*	92	4.262	0	0
6	*Arthritis Res Ther*	84	4.302	0	0
7	*Arthritis Care Res*	82	3.731	0	0
8	*Curr Opin Rheumatol*	73	5.191	0	0
9	*Semin Arthritis Rheum*	73	3.806	0	0
10	*Lupus*	68	2.783	0	0
11	*Clin Exp Rheumatol*	62	2.655	0	0
12	*Rheum Dis Clin North Am*	61	2.096	0	0
13	*Best Pract Res Clin Rheumatol*	58	3.55	0	**1**
14	*Nat Rev Rheumatol*	52	9.745	0	0
15	*Scand J Rheumatol*	52	2.216	0	0
16	*Clin Rheumatol*	52	2.037	0	**5**
17	*Joint Bone Spine*	43	2.748	0	0
18	*Rheumatol Int*	43	2.214	**2**	**4**
19	*BMC Musculoskelet Disord*	41	1.875	0	**3**
20	*Curr Rheumatol Rep*	37	-	0	0
21	*Z Rheumatol*	31	0.45	0	0
22	*J Clin Rheumatol*	29	1.183	0	**1**
23	*Mod Rheumatol*	27	1.716	0	0
24	*J Musculoskelet Pain*	25	0.328	0	0
25	*Reumatismo*	13	-	0	0
26	*Int J Rheum Dis*	12	1.65	0	0
27	*Musculoskeletal Care*	12	-	0	0
28	*Pediatr Rheumatol Online J*	10	1.469	0	0
29	*Rev Bras Reumatol*	10	0.864	1*	1*
30	*Acta Reumatol Port*	10	0.695	0	0
31	*Reumatol Clin*	7	-	0	0
32	*Reumatizam*	4	-	**2**	0
33	*Open Rheumatol J*	3	-	0	0
34	*Ther Adv Musculoskelet Dis*	2	-	0	0
35	*Case Rep Rheumatol*	-	-	0	0
36	*ISRN Rheumatol*	-	-	0	0
37	*Int J Rheumatol*	-	-	0	0

*Revista Brasileira de Reumatologia published a note on duplicate submission and disputed corresponding authorship of one research paper from Sudan, which is currently on hold (15). The journal *h*-index values are picked from the SCImago database (as of January 30, 2014). The 2-Year Journal Impact Factor (2-Y JIF) values are recorded based on Journal Citation Reports^®^ (2013 edition).

**Table 5:** Retracted articles in rheumatology journals (as of January 30, 2014)

Ref.	Journals	Article types	Countries	Publication	Retraction	Reasons for retraction	Cites in Scopus
(16)	*Arthritis Rheum*	Open-label trial	Belgium	2001	2013	Methodologic errors	121
(17)	*Arthritis Rheum*	Followup study	Belgium	2002	2013	Methodologic errors	61
(18)	*Arthritis Rheum*	Observation	UK	2002	2006	Incorrect presentation of scientific data	119
(19)	*Arthritis Rheum*	Observation	Japan	2007	2008	Data falsification	7
(20)	*Arthritis Rheum*	Review	Netherlands	2010	2011	Withdrawal at the authors’ request	0
(21)	*Ann Rheum Dis*	Review	UK	2005	2009	Duplicate publication	38
(22)	*Ann Rheum Dis*	Cohort study	USA	2008	2008	Duplicate publication	0
(23)	*Rheumatology (Oxford)*	Meta-analysis	China	2011	2011	Errors in data extraction and statistical analyses	6
(24)	*Best Pract Res Clin Rheumatol*	Review	USA	2004	2008	Plagiarism	7
(25)	*Clin Rheumatol*	Review	Italy	2009	2012	Plagiarism	8
(26)	*Clin Rheumatol*	Editorial	Italy	2010	2011	Plagiarism	0
(27)	*Clin Rheumatol*	Review	Italy	2010	2011	Plagiarism	8
(28)	*Clin Rheumatol*	Review	Italy	2010	2011	Plagiarism	12
(29)	*Clin Rheumatol*	Questionnaire validation study	Argentina	2012	2013	Copyright infringement	0
(30)	*Rheumatol Int*	Cohort study	Turkey	2011	2012	Statistical error	0
(31)	*Rheumatol int*	Cohort study	Turkey	2011	2012	Statistical error	2
(32)	*Rheumatol int*	Cohort study	Turkey	2011	2012	Statistical error	0
(33)	*Rheumatol int*	Review	Italy	2011	2012	Plagiarism	0
(34)	*BMC Musculoskelet Disord*	Cross-sectional survey	Australia	2010	2011	Absence of institutional ethics approval	4
(35)	*BMC Musculoskelet Disord*	Randomized trial	Australia	2010	2011	Absence of ethics approval	7
(36)	*BMC Musculoskelet Disord*	Cross-sectional survey	Australia	2009	2011	Absence of ethics approval	11
(37)	*J Clin Rheumatol*	Randomized trial	China	2010	2011	Plagiarism and fabrication	22
